# Can Sophie's Choice Be Adequately Captured by Cold Computation of Minimizing Losses? An fMRI Study of Vital Loss Decisions

**DOI:** 10.1371/journal.pone.0017544

**Published:** 2011-03-11

**Authors:** Qi Li, Shaozheng Qin, Li-Lin Rao, Wencai Zhang, Xiaoping Ying, Xiuyan Guo, Chunyan Guo, Jinghong Ding, Shu Li, Jing Luo

**Affiliations:** 1 Institute of Psychology, Chinese Academy of Sciences, Beijing, China; 2 Graduate University of the Chinese Academy of Sciences, Beijing, China; 3 Learning & Cognition Lab, Capital Normal University, Beijing, China; 4 Department of Psychiatry and Behavioral Sciences, Stanford University School of Medicine, Stanford, California, United States of America; 5 Center for Social Psychology Studies, Institute of Sociology, Chinese Academy of Social Science, Beijing, China; 6 Department of Psychology, Fudan University, Shanghai, China; The University of Melbourne, Australia

## Abstract

The vast majority of decision-making research is performed under the assumption of the value maximizing principle. This principle implies that when making decisions, individuals try to optimize outcomes on the basis of cold mathematical equations. However, decisions are emotion-laden rather than cool and analytic when they tap into life-threatening considerations. Using functional magnetic resonance imaging (fMRI), this study investigated the neural mechanisms underlying vital loss decisions. Participants were asked to make a forced choice between two losses across three conditions: both losses are trivial (trivial-trivial), both losses are vital (vital-vital), or one loss is trivial and the other is vital (vital-trivial). Our results revealed that the amygdala was more active and correlated positively with self-reported negative emotion associated with choice during vital-vital loss decisions, when compared to trivial-trivial loss decisions. The rostral anterior cingulate cortex was also more active and correlated positively with self-reported difficulty of choice during vital-vital loss decisions. Compared to the activity observed during trivial-trivial loss decisions, the orbitofrontal cortex and ventral striatum were more active and correlated positively with self-reported positive emotion of choice during vital-trivial loss decisions. Our findings suggest that vital loss decisions involve emotions and cannot be adequately captured by cold computation of minimizing losses. This research will shed light on how people make vital loss decisions.

## Introduction

Organisms follow the principle of approaching gains and avoiding losses for survival and thus act as “adaptable executors” [1]. Compared to gains, losses have a greater impact on survival [2], [3]. Some studies have found that people often place more emphasis on losses relative to gains of equivalent value in risk choice [4], [5] and intertemporal choice [6]. Thus, the study of loss decisions is particularly important for our survival.

The strategy of “sacrificing a pawn to save a rook” is a sensible and effective way to react to existential threats in both animals and human beings. Research in animals has shown that when confronting fatal threats, animals usually choose to sacrifice part of their body to prevent loss of life. For example, the house lizard will sacrifice its tail and run away quickly to survive [7], and the sea cucumber ejects its visceral organs to escape harm from predators [8]. These examples, and many others, demonstrate the willingness of animals to sacrifice a part of their bodies to save their own lives. Human beings can also make dramatic sacrifices, not only to preserve their own lives, but also to preserve other vital things, such as health, nature, love, honor, justice or human rights, each of which can be regarded as distinctly important, absolute, inviolable, and non-negotiable [9]–[11]. These things can be considered irreplaceable, and their loss may present a greater threat to the survival and reproduction of human beings than the loss of ordinary objects, such as money [12], [13]. Hence, exploring the neural basis of vital loss decisions is particularly important from an evolutionary perspective.

The movie *Sophie's Choice* presents two vivid examples of how vital the loss decision is and how the strategy of “sacrificing a pawn to save a rook” is used. Sophie was first asked to choose which of her children would live and which would die. This choice is extremely hard for her because, regardless of her decision, she will lose one of her children. However, she had been told that if she did not choose, both children would be sent to die. Second, when she is forced to accept either her own rape or the loss of her son, Sophie chooses her own rape to save her son's life and, afterwards, was glad to see that the Nazi marshal agreed not to kill her son.

In the broad class of alternative-based choice models, choices among options are presumed to be guided by a principle of value maximization [14]; i.e., the options are independently assigned an overall value, these values are compared, and the option with the highest value is chosen [15], [16]. This assumption seems very simple and straightforward because decision-makers are only required to assign a subjective value or utility to each option and then choose the option that maximizes gains or minimizes losses. These lines of research have one thing in common: the difficulty in arriving at a choice is determined by the similarity of alternatives in subjective value or utility [17], [18], and the influence of emotions on decision making is largely ignored.

How is the principle of value maximization (minimizing losses) played out in Sophie's Choice? On one hand, according to this principle, people are only required to compute the overall value or utility of each alternative and then choose the option that minimizes losses. The more similarity that exists between the overall values or utilities of each alternative, the harder the decision becomes [17], [18]. In Sophie's case, the decision's difficulty stemmed from the similar overall values of each alternative (i.e., the lives of her son and her daughter are equally important). Following the same logic, the difficulty of making a hypothetical choice between losing a male puppet and a female puppet should be similar (i.e., a male puppet and a female puppet are equally important). However, it is obvious that the former decision is much harder than the latter and that it is accompanied by a spontaneous strong emotion. On the other hand, whether Sophie chooses to sacrifice her son or her own morality, she will incur an absolute loss without any gain [19]. The assumption of cold computation cannot explain why Sophie was glad after making a choice that resulted in an absolute loss. The two decisions Sophie made cast doubt on whether cold computation is valid for describing the vital loss decisions people face.

The mechanisms underlying vital loss decision-making remain largely undefined. To address this gap in understanding, we designed an fMRI study in which we asked participants to make forced choices between two losses. We investigated neural responses across three loss conditions: (i) vital-vital loss decision (VV), in which the two options are both vital; for example, losing eyes and losing legs; (ii) vital-trivial loss decision (VT), in which one of the two options is trivial while the other is vital; for example, losing a table lamp and losing a leg; and (iii) trivial-trivial loss decision (TT), in which the two options are both trivial; for example, losing a table lamp and losing a telegram.

In the present study, we examined two predictions regarding the psychological and neural mechanisms that underlie vital loss decisions. Our first prediction was that people would experience greater conflict, decision difficulty, and threat during VV loss decisions than during TT loss decisions. Research indicates that people usually try to predict the consequences of their decisions in advance when choosing between vital loss decisions where either option poses a great threat to survival. Furthermore, increased severity of the predicted consequences leads directly to an increase in the difficulty of making decisions and in the emergence of negative emotions [20]–[22]. Therefore, compared to TT loss decisions, VV loss decisions would be expected to produce significant activation in the prefrontal cortex (PFC), which is involved in the processes of abstract reasoning and cognitive control [23], and in the rostral anterior cingulate cortex (rACC), which is associated with emotional conflict [24], [25] and negative utility [26]. In addition, the amygdala, a more primitive area sensitive to threatening information and negative emotion, would be predicted to become activated [27], [28].

Our second prediction was that people would experience a relative gain accompanied by positive emotion during the VT loss decision. Neuroimaging research on decision-making has shown that if the outcome not chosen is worse than the chosen outcome, increased activation is observed in the orbitofrontal cortex (OFC) and striatum, two putative reward-related areas [29]–[32]; this result indicates that one experiences a relative gain accompanied by positive emotion, which is termed relief [29], [30], [33], [34]. We therefore expect that, during VT loss decisions, people would experience rewarding emotions as a result of contrasting the chosen and unchosen alternatives and would display increased activation in the OFC and striatum.

## Results

### Behavioral results

#### Reaction times, self-reported difficulty and subjective affect ratings

**Figure 1:**
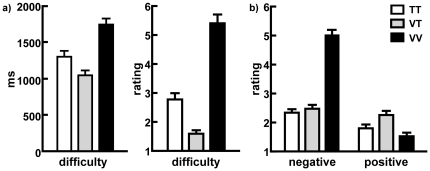
Response Times and Self-Reported Ratings. a) Mean response time (in milliseconds) and self-reported difficulty ratings of choice in each loss decision condition. b) Self-reported negative and positive emotion ratings of choice in each loss decision condition.

The mean reaction times (RTs), self-reported difficulty and positive and negative affect ratings of loss decisions during scanning are shown in Figure 1 for three different conditions. Separate repeated-measures one-way analysis of variances (ANOVAs) were conducted to analyze the average RTs, self-reported difficulty of choice, and negative and positive affect ratings of choice under three loss decision conditions. The results revealed significant main effects of condition in RTs (F(2, 52) = 80.01, *p*<0.001), difficulty of choice (F(2, 52) = 80.91, *p*<0.001), negative affect ratings of choice (F(2, 52) = 106.42, *p*<0.001) and positive affect ratings of choice (F(2, 52) = 9.25, *p*<0.01). Further simple effects analysis showed that the difference in RTs was significant (*p*<0.001): the fastest response to VT, then to TT and the slowest one to VV. The difference in the ratings of choice difficulty was significant (*p*<0.001): VV was rated highest, then TT and finally VT. Furthermore, negative affect ratings of choice in the VV condition were significantly greater than those in the TT and VT conditions (all *p* values<0.001), but no significant difference was observed between the TT and VT conditions. The positive affect rating of choice in the VT condition was significantly greater than in the TT and VV conditions (all *p* values<0.01), but no significant difference was observed between the TT and VV conditions.

### Imaging results

#### Neural activation related to vital-vital loss decisions

**Figure 2:**
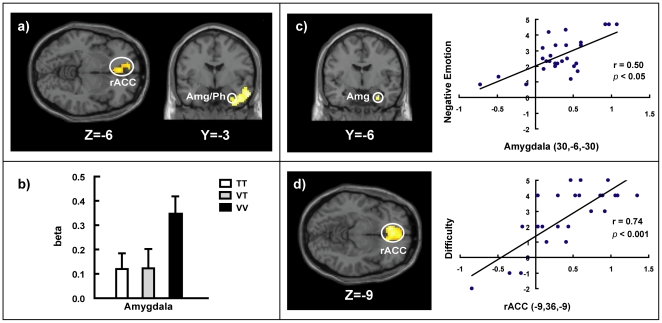
Response Related to Vital-Vital Loss Decisions. a) Selected brain regions showing significant activations when contrasting VV with the TT condition by controlling reaction time, including rACC and amygdala/parahippocampal gyrus. b) The bar graph displays the estimated beta values at the bilateral amygdala of an anatomically defined mask, extracted under the TT, VT, and VV conditions. c) Activation in the amygdala correlated positively with self-reported negative emotion of choice in the contrast of VV minus TT condition. The scatter plot is for illustrative purposes. d) Activation in the rACC correlated with self-reported difficulty of choice in the contrast of VV versus TT condition. The scatter plot is for illustrative purposes. Each point represents the data from a single participant. Notes: rACC, rostral anterior cingulate cortex; Amg, amygdala; Ph, parahippocampal gyrus.

To identify the brain regions specifically associated with VV loss decisions, we compared the VV condition to the TT condition. The results revealed significant clusters distributed in a wide network, including the medial PFC, rACC, anterior medial temporal lobe (MTL) extending into the amygdala, and posterior cingulate gyrus (PCC) (Table 1). To mitigate potential confounds of difficulty between VV and TT conditions, we performed further analysis by treating the difference in RTs between these two conditions as a covariate. This analysis yielded the same results (Figure 2a), indicating that our observed activations were specifically associated with vital loss decisions rather than difficulty.

**Table 1:** Brain activations in VV vs. TT conditions.

Brain region	BA	MNI coordinates			Z-value	Cluster, K_E_
		x	y	z		
L. medial PFC	8	−9	57	39	6.31	2052
R. medial PFC	9	6	54	21	4.9	
L. rACC	32	−15	51	0	4.74	
	24	−3	36	−6	3.65	
R. anterior MTL	21	57	0	−27	5.44	702
	38	39	18	−42	5.17	
R. parahippocampal gyrus	35	24	−9	−33	4.22	
R. amygdala	-	30	−9	−15	3.26	
L. sub-gyral	-	−39	−36	−3	5.41	2049
L. superior temporal gyrus	39	−36	−51	27	5.29	
L. PCC	31	−3	−51	33	4.74	319

Notes: Only clusters (with local maxima coordinates) up to the threshold of *p*<0.05 correction with 20 or more contiguous voxels were reported.

To further investigate whether activation in the amygdala differed among the three different conditions, we first performed an F contrast reflecting the main effect of loss decision conditions. We found significant clusters in the bilateral amygdala [local maxima at (30, −9, −15), (−30, −9, −15), cluster *p*<0.05 small volume correction (SVC)]. We therefore performed region of interest (ROI) analysis for the bilateral amygdala, revealing a significant main effect of loss decision conditions (F(2, 52) = 3.82, *p*<0.05). Further simple effect analyses showed that the activation of the amygdala in the VV condition was significantly greater than the TT (*p* = 0.07) and VT (*p*<0.05) conditions, but no significant difference was observed between TT and VT conditions (Figure 2b).

Moreover, to investigate whether neural activation of vital loss decisions (i.e., VV versus TT conditions) correlated with negative emotion and difficulty, we conducted two separate simple regression analyses on the whole brain with either the difference in self-reported negative affect ratings or difficulty between these two conditions as a covariate of interest. We found that activation in the amygdala [local maxima at (30, −6, −30), cluster *p*<0.05 SVC; Figure 2c] and the PCC [local maxima at (6, −57, 27), (−6, −51, 24), cluster *p*<0.05 corrected] was positively correlated with an individual's negative affect ratings of decision making, whereas the medial PFC [local maxima at (−12, 54, 42), cluster *p*<0.05 corrected] and rACC [local maxima at (−9, 36, −9), (6, 54, 0), cluster *p*<0.05 corrected; Figure 2d] were positively correlated with the difficulty rating of decision making.

#### Neural activation related to vital-trivial loss decisions

**Figure 3:**
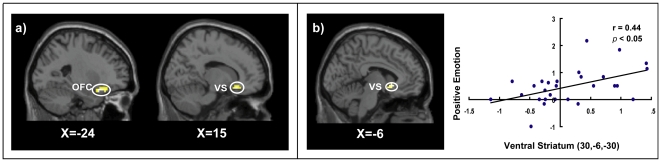
Response Related to Vital-Trivial Loss Decisions. a) Selected brain regions showing significant activation when contrasting VT with TT condition, including the OFC at (−24, 18, −18) and the ventral striatum at (15, 24, −15). b) Activation in the ventral striatum correlated with self-reported positive emotion of choice in the contrast of VT minus TT condition. Scatter plots are for illustrative purposes. Each point represents the data from a single participant. Notes: OFC, orbitofrontal cortex; VS, ventral striatum.

To identify the brain regions specially associated with vital-trivial loss decisions, we performed a contrast of VT versus TT conditions. The results revealed significant activation in the OFC and ventral striatum (Figure 3a, Table 2). To mitigate potential confounds of difficulty between VT and TT conditions, we performed further analysis by treating the difference in RTs between these two conditions as a covariate. This analysis yielded the identical results, indicating that our observed activations were specifically associated with vital loss decisions. To further investigate whether neural activation under VT condition (versus TT condition) correlated with positive emotion and RTs, we conducted separate simple regression analyses with either self-reported positive emotion scores or RTs as a covariate of interest. The results revealed that the ventral striatum [local maxima at (−6, 15, −6), cluster *p*<0.05 corrected] was positively correlated with self-reported positive emotion of decision-making (Figure 3b). The caudate body [local maxima at (−21, −18, 30), cluster *p*<0.05 corrected] and the caudate tail [local maxima at (33, −39, 6), cluster *p*<0.05 corrected] were positively correlated with RTs.

**Table 2:** Brain activations in VT vs. TT conditions.

Brain region	BA	MNI coordinates			Z-value	Cluster, K_E_
		x	y	z		
L. OFC	47	−24	18	−18	4.67	57
L. ventral striatum	-	−12	21	−15	3.51	
R. ventral striatum	-	15	24	−15	4.3	34
R. OFC	47	24	18	−18	3.38	
L. middle temporal gyrus	38	−36	3	−45	4.13	38
L. superior temporal gyrus	38	−42	18	−45	4.05	

Notes: Only clusters (with local maxima coordinates) up to the threshold of *p*<0.05 correction with 20 or more contiguous voxels were reported.

## Discussion

At the time of this publication, very little research existed in which the neural mechanisms underlying loss decisions were investigated [35]. To our knowledge, this is the first study to explore the neural mechanisms of vital loss decisions that are closely connected with the survival of human beings. Compared to TT loss decisions, we found that (i) when VV loss decisions were made, increased activation occurred in the medial PFC, rACC, and amygdala, and (ii) during VT loss decisions, increases in brain activity were observed in the OFC and ventral striatum. These findings generally supported our predictions.

### Vital-vital and trivial-trivial loss decisions are equally difficult in “cold” computation but not equally stimulated in “hot” emotion

Our analyses of the behavioral and neuroimaging data reveal that cold computation of minimizing losses cannot adequately describe VV loss decisions for several reasons.

First, according to previous studies, the similarity of alternatives determines the choice difficulty [17], [18]. In both the TT and VV loss decisions, the decision difficulty in cognitive effort should be the same as a result of the similarity of each alternative. However, behavioral data indicated that both the objective RTs and the subjective self-reported choice difficulty were increased when making VV loss decisions compared to when making TT loss decisions. The VV loss decisions resulted in increased self-reported choice difficulty and brain activation in the medial PFC and rACC. Notably, the medial PFC is important for cognitive control, and its activity reflects the cognitive effort of decision making [23], while the rACC is involved in conflict resolution, and its activity reflects hesitation during decision making [24]–[26]. The behavioral and neural difference in decision difficulty between VV and TT loss decisions cannot be accounted for by the assumption of “cold” computation, suggesting that the similarity of alternatives determines choice difficulty.

Second, our behavioral data revealed that the self-reported negative emotion related to choice in VV loss decisions was greater than in TT loss decisions. The self-reported negative emotion related to choice increased with increased activation in the typical emotion-related areas, including the amygdala and PCC. These activated areas process emotional experiences and emotion-related memories [28], [36]. Particularly, the amygdala is a well-known emotion-related area and is sensitive to highly negative and threatening information [27]. These results suggest that VV loss decisions engaged emotional processing. In addition, the neural network in our study was different from those networks observed in task difficulty studies. Compared to the TT loss decisions, the VV loss decisions showed stronger activation of emotion-related areas, including the medial prefrontal cortex, rACC, and amygdala, even when controlling for the difference in RTs between conditions. In contrast, converging evidence showed that task difficulty (i.e., the complexity of the cognitive process) could activate a set of typically cognitive subdivisions, including the dorsolateral prefrontal cortex, dorsal anterior cingulated cortex, and parietal cortex [37]–[40]. Our findings, together with the evidence obtained from cognitive difficulty, suggested that the emotions elicited by VV loss decisions were not generally engaged during difficult decisions but were dependent on content. The engagement of emotion in VV loss decisions provides behavioral and imaging evidence to question whether a person's vital loss decisions are based solely on a cold computation process.

It is still unclear why decision difficulty differs between VV and TT loss decisions. One possibility is that decision makers realize that something particularly important and delicate is at stake when making VV loss decisions [41]. They may know that the loss of more important things generates a greater threat to their survival. In order to avoid this threat to their survival, people may hesitate to make decisions and strongly experience the negative emotion [20]–[22]. Alternatively, emotions may play an “advisory” or “informational” role in decision making [33], [41], [42]; they may remind decision makers to protect the significant things that can seriously threaten their survival if lost [33], [41]–[43]. In VV loss decisions, the negative emotion aroused by being afraid to choose to lose vital things may also serve as a reminder to be prudent when making decisions.

It is worth noting that the brain activity pattern of VV (difficult) versus TT (easy) conditions is very similar to that of personal moral judgment (difficult) versus impersonal moral judgment (easy) in previous studies [43]–[45]. Studies on both moral judgments and vital loss decisions found a longer reaction time and a greater activation of emotion-related areas (e.g., MPFC, PCC, and amygdala) in more difficult conditions (i.e., VV condition and personal moral judgment condition). We maintain that from a psychological point view, the crucial similar consequences between our vital loss decisions and previous personal moral judgments [41], [43]–[45] lie in the engagement of people's emotions. However, our study differed from previous personal moral judgments in a number of ways. First, vital loss decisions involved the self-referenced decisions, in which decision makers authored serious harm to themselves. In contrast, moral judgments were associated with the other-referenced decisions, in which decision makers authored serious harm to others. Second, in vital loss decisions, emotions were negatively aroused because people's choice was contrary to a human's innate goal of survival, whereas, in moral judgments, the emotions were negatively aroused because people's choice was contrary to their moral and ethical principles that have been formed through interaction with society. Thus, the present study extends previous findings in emotionally difficult decisions.

### Vital-trivial versus trivial-trivial loss decisions: a surprising reward appears from pure losses

The analysis of the behavioral and neuroimaging data revealed that VT loss decisions were not well described by cold computation of minimizing losses. Interestingly, when there was a choice between two losses in VT and TT loss decisions, trivial options were definitely lost in both conditions, but the emotion accompanying a chosen loss was more positive in VT loss decisions. Specifically, behavioral data revealed that compared to TT loss decisions, participants' self-reported positive emotion was greater when making VT loss decisions. Imaging data revealed that activation in the ventral striatum increased with increasing self-reported positive emotion of choice. The ventral striatum is correlated with reward responses [31], pleasant stimuli [46], and the extinction of fear conditioning [47]. Similar findings have been reported in studies using monetary [30] and shock [29] stimuli, which have shown similar emotional responses (i.e., happiness) with relief-eliciting positive relative values and joy-eliciting positive absolute values. Our findings, together with the evidence obtained from investigating non-vital choices, suggest that in VT loss decisions, the reward-related emotion of relative gains is experienced when the decision maker contrasts the chosen and unchosen outcomes. These findings pose a challenge for the assumption that loss decisions are simply based on a “cold” loss minimization process.

Why do people experience rewarding feelings when they make a pure loss choice? One possible explanation is that their reference point changes the absolute loss into a relative loss. In their prospect theory, Kahneman and Tversky [48] argued that loss is a relative rather than an absolute concept in VT loss decisions. According to the reference point account, participants may think of losing trivial things (i.e., a less threatening outcome) as gains when compared to the possibility of losing vital things (i.e., a more threatening outcome). In other words, something particularly important can be protected [41], leaving decision makers with feelings of positive emotion. If this is the case, the wise strategy of “sacrificing a pawn to save a rook” may have been inherited over the course of evolution.

An alternative explanation concerning the activation of reward-related areas aroused by being given an easy choice also deserves attention. To test whether this explanation holds, we chose reaction times as an index of easiness. We found that reward-related areas were not positively correlated with reaction time, whereas by controlling the reaction times, the reward-related areas, including the orbital frontal cortex and ventral striatum, remained activated in VT compared to TT loss decisions. Additionally, previous imaging reports have shown that the contrast of easy versus difficult (i.e., the complexity of the cognitive process) activates a set of typically cognitive subdivisions, including the dorsolateral prefrontal cortex (DLPFC), inferior frontal gyrus, superior frontal gyrus, superior temporal gyrus, and middle temporal gyrus [49]–[51]. These brain areas are different from those observed in our VT versus TT loss decisions. This evidence led us to believe that the observed positive emotion was not simply due to the fact that VT choices are easy.

To our knowledge, the neural correlate of positive emotion associated with relative gain is under-studied, and this emotion is a prevalent experience in humans and part of our everyday ordinary lives. Although we did not directly check whether the observed positive emotion was relief, we conjectured that the positive emotion associated with relative gain in our study was something similar to relief because the positive emotion was aroused by having done the right thing when the unchosen outcome was worse than the chosen outcome. What we observed was very similar to relief investigated in previous studies [29], [30], [34]. Future research on the relief perspective is required.

### Limitations and Prospects

One possible limitation of the current study is that it employed hypothetical decision problems, although the participants were instructed to imagine the real situations. However, it is not possible to force participants to incur any realistic vital loss in order to study vital loss decisions. In addition, converging evidence has shown that emotion can be produced and studied in a hypothetical manner [41], [43], [52]–[56]. For example, emotions can be reliably induced via empathic or hypnotic approaches [54], [56], through the imagination of future negative events [52] or by the subjective imagination of words and phrases conveying painful experiences [53], [55], even though these conditions did not cause any real harm to the subjects. Moreover, the use of hypothetical problem situations has been widely adopted in decision-making studies, such as the footbridge dilemma [45] or a tragic trade-off between “safety at work” and “environmental protection” [41]. Thus, hypothetical situations can be effective in eliciting emotion and investigating the emotional components of decision making [41].

Utility theory has dominated the analysis of medical decision making for decades [57]–[59]. When faced with a decision about medical treatments and health programs that requires trade-offs between quality of life (morbidity) and quantity of life (mortality), physicians and surgeons are often instructed to use the utility analysis technique to provide treatment recommendations to assist their patients in making treatment decisions. Physicians often bear the responsibility of assessing options on the patient's behalf, but they are poor judges of patients' preferences involving vital loss. The quality-adjusted life year (QALY) is currently the most important utility model in medical decision making and has been developed in an attempt to calculate the treatment outcomes into an overall value [58]. Taking Mehrez and Gafni's (1989) study as an example, the utility of treatment A (go through painful medical treatments for a period of three months and then live the rest of the life (ten years) in full health) is equal to 0, which means that going through the treatment is equal to dying; the utility of treatment B (avoid treatment and stay in this current state of health for a shorter period (eight years)) is equal to 0.95 [59]. However, discrepancies between QALY calculations and an individual's own preferences have been reported in previous studies [57]–[59]. An important shift in the provision of health care over the last decade towards patient-centered approaches and shared decision-making has paved the way for the incorporation of patients' preference within the clinical consultation. Therefore, our findings may help in better understanding the discrepancies between QALY calculations and an individual's own preference [57]–[59] and call for a reconsideration of the current framework of medical decision making in patient-doctor communication.

To summarize, our findings suggest that vital loss decisions involve emotion rather than pure cold computation. This research has shed light on the neural mechanisms involved in making vital loss decisions and has provided an improved understanding of how people make vital loss decisions.

## Materials and Methods

### Participants

Twenty-seven young, healthy university students (aged 19 to 28 yr, mean age±SE: 22.41 yr±2.50 yr; 7 males) participated in this study. They reported no neurological or psychiatric history. Informed consent was obtained from all participants prior to the experiment. The procedure was approved by the Institutional Review Board of the Institute of Psychology, Chinese Academy of Sciences.

### Stimuli

Initially, we carefully selected 1,100 high-frequency Chinese nouns consisting of two characters each [60]. The importance and familiarity for each noun were separately rated by a different group of 35 participants on a 7-point scale (1 to 7: unimportant to extremely important; unfamiliar to extremely familiar). In the present study, importance was defined as how desirable or valuable the thing was in each person's life. The final set of stimuli consists of two categories of nouns: 60 vital nouns with high ratings of importance (mean±SE = 5.92±0.04) and 60 trivial nouns with low ratings (mean±SE = 2.71±0.07: *t*(59) = 41.99, *p*<0.001) while maintaining the same familiarity constant (*t*(59)<1). Subsequently, nouns from the two categories were paired in terms of their importance, resulting in three sets of 20 pairs: vital-vital (VV), vital-trivial (VT), and trivial-trivial (TT). The pairings were counterbalanced across the participants.

### Procedure

Prior to scanning, participants completed two specific “loss” examples to maximally involve themselves in the experimental situation. The first trivial example was “*Loss of cola*.” The participants were instructed to drink a small amount of cola and describe its flavor. They were then told that they would not be able to drink the cola again and would never enjoy the taste of cola if they chose to lose it over the other option. The second vital example was “*Loss of eyes*.” The participants were told that they would not have eyes for the rest of their lives if they chose this option. Their eyes were covered with a black cloth, and they were asked to search for a shuttlecock in the room. In this session, the participants were told that this task was neither a game nor an ability test but was being conducted so that they would experience the feeling of losing their eyes.

**Figure 4:**
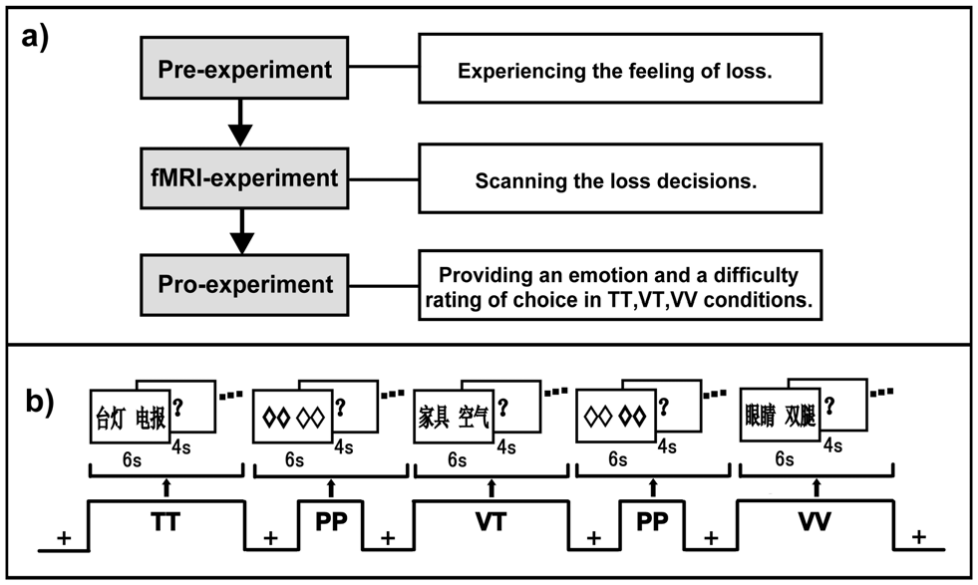
Experimental Design. a) The experiment consisted of three parts. Before scanning, two examples were given to participants to familiarize them with the experimental situation. Then, participants took part in an fMRI experiment in which they made trivial-trivial (TT), vital-trivial (VT), and vital-vital (VV) loss decisions. After scanning, participants were required to rate their emotions and the difficulty of the decision on a 7-point scale outside the scanner. b) An example of different experimental conditions during fMRI scanning. Notes: The Chinese words in the figure, 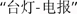, 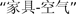, and 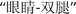 mean “lamp-telegraph”, “furniture-atmosphere”, and “eyes-legs” respectively.

Thereafter, participants underwent a blocked-design functional magnetic resonance imaging (fMRI) experiment while performing three blocks of loss decision tasks including TT, VT, and VV, and two blocks of visual perceptual decision tasks (PP). The PP task was used as a perceptually equalized control baseline condition relative to the other three experimental conditions (i.e., VV, VT, and TT) of interest. These blocks were interleaved by fixation (FX) to gain signal-to-noise contrast in the fMRI studies [61], [62]. The order of these blocks was TT-PP-VT-PP-VV (Figure 4). This order was fixed from low to high to “ease participants in” by not starting with a very hard and highly threatening decision and to decrease the likelihood of Type II errors [63], [64]. In the FX condition, participants were required to passively look at a fixation cross on the screen for 20 s. For the remaining experimental conditions, each block started with an instruction cue for 6 s, followed by 10 trials of a 6-s presentation of paired-items. Trials were separated by a question mark for 4 s. In the PP condition, participants were asked to press the left key if a bold rhombus was on the left and to press the right key if it was on the right. In the three loss decision conditions, participants were instructed to press the left key if they decided to lose the item presented on the left side, and to press the right key for the alternative option when the question mark presented.

Subsequently, participants were asked to retrospectively recollect their emotions and difficulties associated with the decision situation they encountered in the TT, VT, and VV conditions separately. Emotions related to choice were assessed using the Positive and Negative Affect Scale (PANAS), including ten negative and ten positive items separately [65]. Participants were asked to indicate their feelings on 7-point scales ranging from 1 (not at all) to 7 (extremely) (e.g., “afraid”, “jittery”). Levels of choice difficulty were assessed by a 7-point scale separately ranging from 1 (not at all difficult) to 7 (extremely difficult). The order of the two measurements was counterbalanced. To aid their memory retrieval, participants were provided with the list of items they had encountered during the TT, VT, and VV task blocks while they were answering these questionnaires.

### fMRI data acquisition

During MRI scanning, whole brain T2*-weighted echo planar imaging based on blood oxygenation level-dependent contrast (EPI-BOLD) fMRI data was acquired with a Siemens Trio 3.0-T MR-scanner (Erlangen, Germany) using a standard head coil at the Shanghai Key Laboratory of Magnetic Resonance Imaging. Thirty-two transverse slices covering the entire brain were acquired using a gradient-echo echo-planar pulse sequence (repetition time, TR = 2000 ms, echo time, TE = 30 ms, field of view, FOV = 220 mm, flip angle = 90°; matrix 64×64×32, spatial resolution, 3.4 mm×3.4 mm×3 mm). Anatomical images were obtained using a standard 3D T1-weighted sequence (matrix 256×256×128; spatial resolution, 0.938 mm×mm 0.938×1.33 mm, TR = 2530 ms, TI = 1100 ms, TE = 3.98 ms).

### fMRI data analysis

Image preprocessing and statistical analysis were performed using the Statistical Parametric Mapping software (SPM5). Prior to image preprocessing, the first five functional EPI volumes were discarded to allow for T1 stabilization. The remaining functional images were realigned to the first scan to correct for head movement. Subsequently, those images were normalized to a standard EPI template at the Montreal Neurological Institute (MNI) space with a 3 mm×3 mm×3 mm resample voxel size and were smoothed using a Gaussian filter with 8-mm full width half maximum (FWHM).

To assess the neural activity associated with the three experimental conditions of interest, three loss decision conditions were separately modeled using a box-car function and convolved with the canonical hemodynamic response function built into SPM5. The analysis included high-pass filtering using a cutoff of 1/128 Hz and serial correlations correction using a first-order autoregressive (or AR [1]) model.

In the statistical analysis of fMRI data, PP, TT, VT, and VV blocks were initially modeled as four separate regressors on the first individual level. We separately contrasted TT, VT, and VV conditions with PP condition. Subsequently, on the second level analysis, these contrast parameter estimates generated from the individual level were further submitted into a full factorial group analysis (ANOVA) to allow for population inference using a random-effect model. In the whole-brain search, all results from random effect analysis were initially searched with a threshold at *p*<0.001, uncorrected, with a spatial extent of more than 20 continuous voxels. Unless otherwise specified, only clusters significant at *p*<0.05 corrected for multiple non-independent comparisons were reported [66]. Given our prior hypothesis about the amygdala, this region was additionally investigated with a reduced search region of an anatomically defined mask [67] using an SVC procedure. To characterize activation patterns of three loss decision conditions in the amygdala, ROI analysis was performed by extracting parameter estimates from this region and then submitted to further statistical tests in SPSS (15.0, SPSS Inc, Chicago).
